# Species-specific control of external superoxide levels by the coral holobiont during a natural bleaching event

**DOI:** 10.1038/ncomms13801

**Published:** 2016-12-07

**Authors:** Julia M. Diaz, Colleen M. Hansel, Amy Apprill, Caterina Brighi, Tong Zhang, Laura Weber, Sean McNally, Liping Xun

**Affiliations:** 1 Department of Marine Chemistry and Geochemistry, Woods Hole Oceanographic Institution, 266 Woods Hole Rd, Woods Hole, Massachusetts 02543, USA; 2 Skidaway Institute of Oceanography, Department of Marine Sciences, University of Georgia, 10 Ocean Science Circle, Savannah, Georgia 31411, USA; 3 Department of Chemistry, Imperial College London, Imperial College Road, London SW7 2AZ, UK; 4 MOE Key Laboratory of Pollution Processes and Environmental Criteria, College of Environmental Science and Engineering, Nankai University, 38 Tongyan Road, Tianjin 300350, China; 5 School for the Environment, University of Massachusetts Boston, 100 Morrissey Boulevard, Boston, Massachusetts 02125, USA

## Abstract

The reactive oxygen species superoxide (O_2_^·−^) is both beneficial and detrimental to life. Within corals, superoxide may contribute to pathogen resistance but also bleaching, the loss of essential algal symbionts. Yet, the role of superoxide in coral health and physiology is not completely understood owing to a lack of direct *in situ* observations. By conducting field measurements of superoxide produced by corals during a bleaching event, we show substantial species-specific variation in external superoxide levels, which reflect the balance of production and degradation processes. Extracellular superoxide concentrations are independent of light, algal symbiont abundance and bleaching status, but depend on coral species and bacterial community composition. Furthermore, coral-derived superoxide concentrations ranged from levels below bulk seawater up to ∼120 nM, some of the highest superoxide concentrations observed in marine systems. Overall, these results unveil the ability of corals and/or their microbiomes to regulate superoxide in their immediate surroundings, which suggests species-specific roles of superoxide in coral health and physiology.

## Abstract (web summary)

Corals may vary in their ability to regulate reactive oxygen species (ROS) that can influence coral health. Diaz and colleagues conduct *in vivo* measurements of the ROS superoxide at the surface of corals and find substantial species-level variation in superoxide regulation that is independent of bleaching status.

Coral reefs are among the most biologically rich and economically valuable ecosystems on the planet^1^,^2^. However, more than 30% of the world's coral reefs have vanished over the past 35 years largely due to coral bleaching and diseases^3^,^4^ that are triggered by increasing ocean temperatures^5^. Given the present course of climate change and forecasted temperature increases^6^, there is growing concern that coral reef ecosystems will continue to decline rapidly. Indeed, record-breaking ocean warming associated with El Niño from 2014 to 2016 has devastated coral reefs across the world, resulting in the longest mass coral bleaching event ever recorded^7^.

Coral bleaching involves the loss of *Symbiodinium*—essential algal endosymbionts that provide colour, organic carbon and nutrients to the coral host^8^. These algae are critical members of a highly diverse assemblage of microbes (bacteria, archaea, fungi and other protists) comprising the coral holobiont. Some corals may fully recover, or even resist bleaching completely^4^,^9^, which is primarily attributed to the ability of certain groups of *Symbiodinium* to tolerate elevated temperatures^10^,^11^. However, much less is known about the role of coral hosts and their microbiome in bleaching susceptibility, resistance and recovery. To predict and mitigate future threats to coral reefs across the globe, a more holistic understanding of the processes responsible for maintaining coral health is necessary.

Reactive oxygen species (ROS) play a critical yet enigmatic role in coral bleaching and health. ROS include intermediates in the reduction of molecular oxygen to water, such as the superoxide radical anion (O_2_^·−^). During bleaching, light and heat stress damage the photosynthetic machinery of *Symbiodinium* cells and impair mitochondrial electron transport in the coral host, which is thought to result in the over production of intracellular ROS, the onset of oxidative stress and an antioxidant response throughout the coral holobiont^5^,^12^,^13^. Excessive levels of ROS degrade vital cell components^14^, and superoxide can initiate apoptosis signalling pathways^15^ involved in bleaching and host mortality^16^. However, other potential sources and pathways of ROS production within the coral holobiont have recently been identified. For instance, a wide diversity of heterotrophic bacteria enzymatically produce extracellular superoxide in the dark, including representative isolates of *Roseobacter*, *Vibrio* and other genera commonly found in coral microbiomes^17^. Furthermore, two *Symbiodinium* isolates representing clades A and C produce extracellular superoxide even in the absence of heat and light stress, potentially via transmembrane oxidoreductases, such as NADPH oxidase^18^. In fact, NADPH oxidases were recently implicated as a source of superoxide at the surface of the coral *Stylophora pistillata* in aquaria incubations under non-stressful conditions^18^.

Although the buildup of internal ROS can lead to oxidative stress, external production of superoxide may have positive impacts on coral health. For instance, coral-derived NAD(P)H oxidoreductases putatively involved in the production of extracellular superoxide are associated with increased thermotolerance of the coral *Acropora millepora*^19^ and resistance to pathogenic white band disease in *Acropora cervicornis*^20^. In addition, extracellular superoxide dismutase (SOD) is a necessary virulence factor of the pathogen *Vibrio shiloi*, which causes bleaching in the coral *Oculina patagonica*^21^, thus pointing to coral-derived superoxide as a potential means of resisting pathogens. Given the known role of superoxide in cell signalling, differentiation and proliferation^22^,^23^,^24^, growth promotion^25^,^26^, defence^27^,^28^,^29^ and acquisition of the micronutrient iron^30^,^31^ in many macro- and microorganisms, extracellular production of superoxide may have other benefits to coral health as well. Overall, previous research suggests that the potential origins of superoxide in the coral holobiont are diverse, and biologically controlled levels of superoxide production by corals may be an integral component of coral physiology and immune defence, as seen in higher eukaryotes^27^.

Both intracellular and extracellular superoxide production are clearly important to maintaining the redox homoeostasis and health of corals. Despite the vast array of possible superoxide sources identified in corals, however, the actual origins, distributions and ecological underpinnings of superoxide production in natural coral communities remain largely unknown due to a lack of direct superoxide measurements. Indirect evidence of oxidative stress in bleaching corals is based on observations of antioxidant activity, gene expression and proteomic profiles, yet the methodologies available for directly measuring intracellular ROS are invasive and artifact prone^32^,^33^,^34^, making *in vivo* measurements of ROS difficult. To advance our understanding of superoxide dynamics in the coral holobiont, we capitalized on recent advances in non-invasive chemiluminescent techniques to make the first *in situ* measurements of external superoxide production by several species of thermally stressed and bleaching corals in a natural reef environment.

The goal of this study was to determine whether and to what degree various coral species produce external superoxide on a natural reef and to assess the potential role of coral symbionts in this superoxide production. Results revealed significant species-level control of external superoxide concentrations by the corals *Fungia scutaria*, *Montipora capitata*, *Pocillopora damicornis*, *Porites compressa* and *Porites lobata*. Superoxide concentrations at coral surfaces could not be explained by abiotic photo-driven mechanisms of ROS production, photosynthesis, bleaching status or *Symbiodinium* abundances. Extracellular superoxide production by bacterial symbionts and asymbiotic coral larvae was confirmed in laboratory experiments, supporting the conclusion that superoxide production at the coral surface may originate from the activity of epibionts or the coral host itself.

## Results

### Superoxide production by corals is species-specific

**Figure 1:**
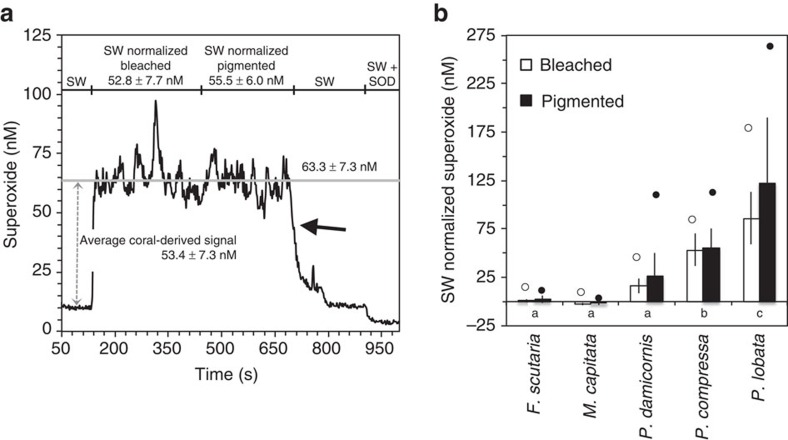
Superoxide produced by bleached and pigmented colonies of field-based corals. (**a**) Representative FeLume trace showing superoxide concentrations millimetres above the surface of a *Porites lobata* colony that had both bleached and pigmented sections. Superoxide data were collected over time by positioning the sample tubing at a static location over the bleached section of the coral for several minutes, and then moving the tubing to a single location over the pigmented section for a similar amount of time. Chemiluminescence signals were converted to superoxide concentrations by first subtracting out signals of an aged filtered seawater baseline (not shown), and then corrected signals were converted to concentrations using the daily calibration curve. The specific coral-derived signal (dashed arrow) was then determined by subtracting the signal obtained at the coral surface from the seawater signal obtained 5–15 cm away from the coral. The average superoxide concentrations between bleached and pigmented sections are not significantly different, as revealed by a two-sample *t*-test (*P*>0.05). Once the tubing was removed from the coral surface, superoxide concentrations rapidly declined back to background seawater (SW) levels (solid arrow). Finally, the addition of SOD, which selectively degrades superoxide, confirmed that chemiluminescence signals were attributable to superoxide. Superoxide concentrations are reported as average±s.d. of the temporal signal. (**b**) Superoxide levels measured for bleached and pigmented colonies of each coral species corrected for background SW concentrations. Circles indicate peak superoxide levels measured for each specimen. For *M. capitata*, superoxide concentrations at the surface of the colony were lower than the background SW, resulting in a negative SW-normalized superoxide concentration. Average species-specific superoxide levels not connected by the same letter (indicated on the x-axis below the bars) are significantly different (*P*<0.05). Error bars indicate s.d., *n*=2 (*F. scutaria* bleached), *n*=2 (*F. scutaria* pigmented), *n*=10 (*M. capitata* bleached), *n*=8 (*M. capitata* pigmented), *n*=13 (*P. compressa* bleached), *n*=9 (*P. compressa* pigmented), *n*=9 (*P. damicornis* bleached), *n*=9 (*P. damicornis* pigmented), *n*=13 (*P. lobata* bleached) and *n*=16 (*P. lobata* pigmented).

During a bleaching event in the Hawaiian Islands in October 2014, a broad range of superoxide concentrations were measured at the surfaces of five coral species in Kaneohe Bay (Supplementary Fig. 1). Background superoxide levels within the reef seawater located at the same depth as the corals but >10 cm from their surface ranged from 4 to 11 nM—values consistent with previously reported superoxide levels in productive marine waters but up to several orders of magnitude higher than in typical open ocean sites^35^,^36^,^37^,^38^,^39^. Average superoxide concentrations measured only millimetres above coral surfaces ranged from levels below bulk seawater (*M. capitata*) to steady-state concentrations that were ∼120 nM higher than bulk seawater (*P. lobata*) (Fig. 1, Supplementary Table 1). These superoxide concentrations are among the highest reported in marine systems yet are consistent with the ability of organisms to substantially increase superoxide concentrations in seawater. For example, in previous aquaria studies, the corals *Stylophora pistillata* and *Porites astreoides* increased seawater superoxide concentrations from ∼2 to 20–35 nM (ref. 18) and from ∼1 to 35 nM (ref. 40), respectively. Furthermore, up to ∼33 nM superoxide was detected in the deep chlorophyll maximum at the subtropical front east of New Zealand^35^. In addition, the most prolific microbial producer of extracellular superoxide, the toxic bloom-forming alga *Chatonella marina*, can produce steady-state concentrations of superoxide reaching 140 nM at bloom-level cell densities *in vitro*^41^.

Full factorial ANOVA was conducted to compare the effects of coral species and health state (bleached versus pigmented) on superoxide concentrations. This analysis revealed that average coral-derived superoxide levels were significantly different as a function of coral species (*F*_4,81_=34.8, *P*<0.01; Fig. 1b). However, health state had no effect on average superoxide levels overall (*F*_1,81_=1.27, *P*=0.26) or when health was separately compared for each species (*F*_4,81_=1.15, *P*=0.34; Fig. 1b). Average superoxide levels were also significantly different in seven out of ten interspecies comparisons using Tukey's honest significant difference test (*P*<0.05; Fig. 1b). Only the species with the lowest superoxide levels were statistically similar, specifically *F. scutaria* and *M. capitata* (*P*=∼1.0), *F. scutaria* and *P. damicornis* (*P*=0.83) and *M. capitata* and *P. damicornis* (*P*=0.24; Fig. 1b).

**Figure 2:**
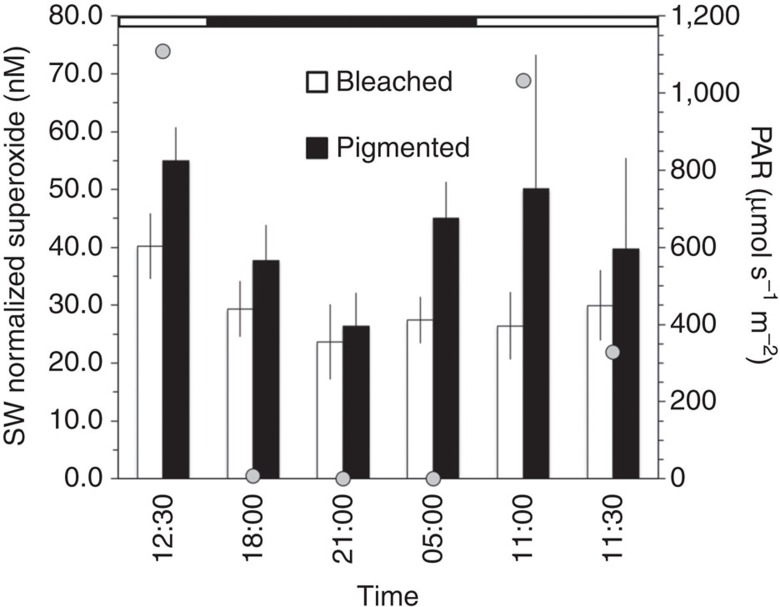
**Diel variability in superoxide concentrations produced by** ***Porites compressa***. To normalize coral-derived superoxide concentrations, superoxide levels in background seawater (SW) at 5–15 cm from the coral surface were subtracted. PAR values are indicated as grey circles. Periods of daylight (white) and darkness (black) are indicated in the horizontal scale at the top of the chart. Error bars indicate s.d. of the temporal signal collected at a rate of 0.5 points per second (*n*=21–124 points).

**Figure 3:**
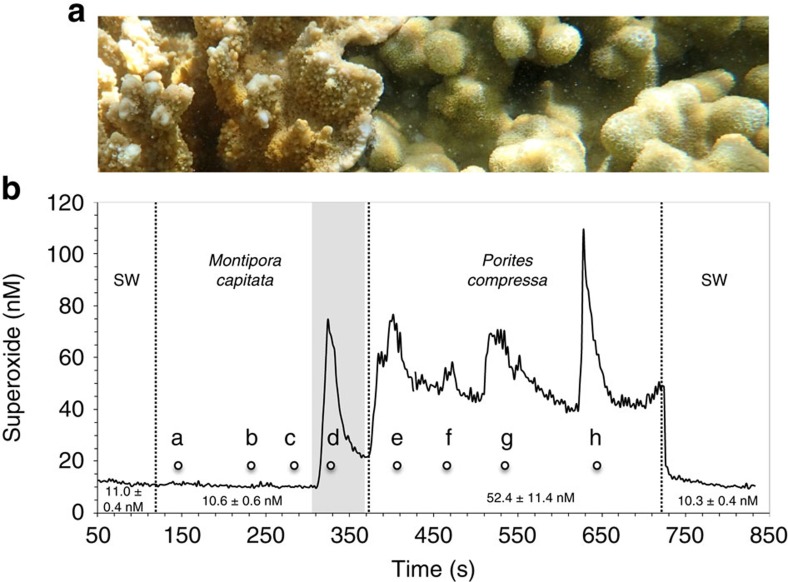
**Superoxide concentrations across adjoining** ***M. capitata*** **and** ***P. compressa***. (**a**) Close-up photo of adjoining *M. capitata* (left) and *P. compressa* (right). (**b**) Continuous superoxide measurements were made by moving the sample tubing across the coral surfaces, pausing for 20–60 s at the following positions: along the *M. capitata* surface at distances of (a) 10 cm, (b) 5 cm, (c) 1 cm and (d) <1 cm (shaded area) from the species interface; and along the *P. compressa* surface at distances of (e) <1 cm, (f) 1 cm, (g) 5 cm and (h) 10 cm away from the species interface. Chemiluminescence signals were converted to superoxide concentrations by first subtracting out signals of an aged filtered seawater baseline (not shown), and then corrected signals were converted to concentration using the daily calibration curve. The superoxide concentrations shown for the corals include both the coral-derived signal and the seawater (SW) signal (not SW corrected on the trace). Superoxide concentrations for *M. capitata* (averaged from a to c) and *P. compressa* (averaged from e to h) were significantly different (*P*=0.014) based on a two-sample *t*-test and are indicated below the superoxide trace, along with average superoxide levels in the background SW adjacent to each coral. On the basis of a two-sample *t*-test, superoxide levels at the *M. capitata* surface (a–c) were statistically similar (*P*>0.1) to the levels in the background SW, except right at the edge of the species interface (d, shaded area). Superoxide concentrations are reported as average±s.d. of the temporal signal.

A number of lines of evidence confirm that the observed superoxide is derived from the coral holobiont. First, superoxide concentrations at coral surfaces rapidly declined to background seawater levels over short distances away from the corals (for example, a centimetre or less), suggesting that corals are a point source, consistent with the short lifetime of superoxide in these waters (maximum half-life in bulk reef water=∼30 s) (Fig. 1a). Second, the presence of statistically similar superoxide levels at the surface of *P. compressa* colonies over a wide range of photosynthetically active radiation levels (PAR=0–1,109 μmol s^−1^ m^−2^; ANOVA *F*_4,2_=1.13, *P*=0.52; Fig. 2 and Supplementary Table 2) rules out abiotic photo-oxidation mechanisms as a major mode of superoxide generation. Similarly, a recent aquaria-based study also found dark production of extracellular superoxide by another *Porites* species, *P. astreoides*^40^. Lastly, the fact that superoxide levels are species-specific (Fig. 1b), even across adjoining colonies of two species (Fig. 3), further indicates that the superoxide detected was derived from the coral holobiont.

The potential direct and indirect pathways of external superoxide formation by corals are currently unresolved. In addition to superoxide, corals also release dissolved organic carbon^42^,^43^ and hydrogen peroxide^44^ to seawater, both of which could in theory contribute to the indirect formation of superoxide. Yet, the observation that coral-derived superoxide is detectable only several millimetres beyond the coral surface reflects the rapid decay kinetics of superoxide, which is inconsistent with the higher residence times and transport distances for dissolved organic carbon and hydrogen peroxide in relation to the coral surface^44^,^45^. Further, organic carbon fluxes from corals and within coral reefs have a pronounced diel nature^46^,^47^, which is not observed here for superoxide production, suggesting that dissolved organic carbon-derived superoxide is not a dominant contributor to the superoxide concentrations reported herein. Further, given the previously reported ability of corals to generate external superoxide through putative NADPH oxidoreductases^18^, most of the observed superoxide likely originates through direct enzymatic pathways.

The species-level trends observed in average superoxide levels are in line with previous measurements of the ROS hydrogen peroxide, which also demonstrated a broad range of species-specific levels at coral surfaces^44^. Specifically, as we observed for superoxide (Fig. 1), steady-state hydrogen peroxide levels measured in a previous study were high for *Porites* sp. (∼500 nM), intermediate for *Pocillopora* sp. (∼250 nM) and near zero for *Fungia* sp.^44^. Species-specific variability in ROS levels may reflect differences in ROS production, degradation or both. Indeed, heterotrophic bacteria^17^ and corals^44^ have previously been shown to simultaneously produce and degrade extracellular ROS. In fact, corals have been shown to release antioxidants into their surroundings^18^,^40^,^44^,^48^, which also varies widely as a function of coral species^44^. This differential antioxidant release may explain why superoxide concentrations in the direct vicinity of *M. capitata* were lower than the surrounding seawater. Moreover, the presence of stable, non-zero levels of superoxide and hydrogen peroxide at the surfaces of coral species that have the ability to degrade external ROS indicates that these external ROS concentrations are not detrimental. While superoxide levels as low as 1 nM can inactivate certain enzymes^49^, this toxicity threshold may vary depending on the site and mechanism of superoxide production, as well as the biological species.

ROS production by coral endosymbionts can contribute to external fluxes of hydrogen peroxide because hydrogen peroxide readily diffuses through cells^48^. On the other hand, superoxide is a charged and much shorter-lived molecule at physiological pH. On the basis of its low diffusivity, it cannot readily pass across biological membranes^50^ unless they are severely compromised^12^. Even if superoxide could diffuse across biological membranes, its intracellular lifetime (∼μs) and diffusive distance (∼100s of nm)^14^ are too short to explain external superoxide concentrations. Thus, endosymbionts are unlikely to contribute to external superoxide levels simply due to their location within the coral. In particular, *Symbiodinium* cells inhabit the symbiosome inside the host's gastrodermal tissue beneath the coral's outermost (epithelial) cell layer. Despite recent evidence that the symbiosome has a pH of ∼4 (ref. 51), which could allow for diffusion of the protonated form of superoxide into the host, the superoxide anion would again dominate in the coral gastrodermal cells where the pH is ∼7 (refs 51, 52). Superoxide produced by *Symbiodinium* would therefore have to pass several biological membranes, multiple cellular compartments and the external mucus layer to contribute to external superoxide levels at the coral surface.

**Figure 4:**
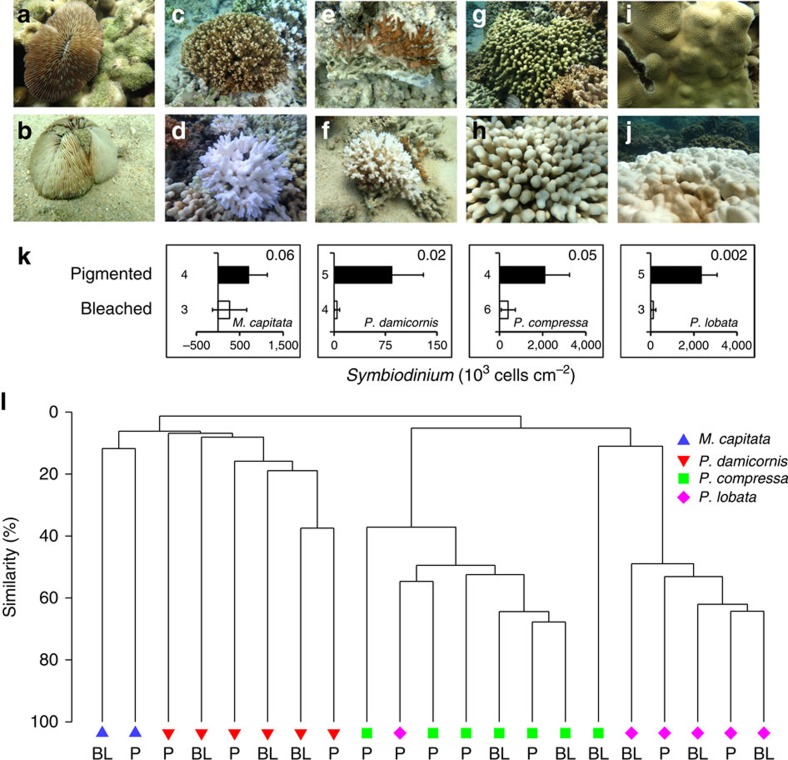
***Symbiodinium*** **cell abundances and microbial community composition in corals.** Representative pigmented (**a**,**c**,**e**,**g**,**i**) and bleached (**b**,**d**,**f**,**h**,**j**) colonies of *F. scutaria* (**a**,**b**), *M. capitata* (**c**,**d**), *P. damicornis* (**e**,**f**), *P. compressa* (**g**,**h**) and *P. lobata* (**i**,**j**). (**k**) Abundance of coral-hosted *Symbiodinium* within pigmented and bleached specimens of each species (except *F. scutaria* because it was not permitted for collection) are reported as average±s.d. *P* values associated with the two-sample *t*-test of average *Symbiodinium* counts for bleached versus pigmented colonies of each species are provided in the upper right corner of each plot. Sample size is indicated next to each bar. (**l**) The bacterial and archaeal communities associated with the corals were found to be similar by coral species rather than by bleaching (BL) or pigmented (P) health state, as indicated by a cluster dendogram of bacterial and archaeal V4 SSU rRNA gene sequences from tissue and mucus samples of coral colonies, compared using Bray–Curtis similarity.

Consistent with the unlikelihood of endosymbionts as a source of external superoxide, several lines of evidence suggest that external superoxide production is decoupled from *Symbiodinium,* even in thermally stressed and bleaching corals. First, *Symbiodinium* were less abundant in bleached versus pigmented coral colonies of the same species (Fig. 4 and Supplementary Table 3), but bleached and pigmented colonies of each species were associated with similar superoxide levels (Fig. 1). In fact, *Symbiodinium* abundances did not correlate significantly with coral-derived superoxide concentrations (Supplementary Fig. 2). Furthermore, superoxide production by *P. compressa* was not significantly different over a diel cycle of PAR levels, even when bleached and pigmented colonies were considered together (ANOVA, *F*_4,2_=1.13, *P*=0.52) or separately (ANOVA, *F*_4,2_=0.23, *P*=0.90) (Fig. 2 and Supplementary Table 2), which precludes photosynthetic mechanisms of superoxide production as a major source. On the basis of these results and the low diffusivity of superoxide, the most likely sources of external superoxide are coral epithelial cells and/or microbial epibionts residing on the coral's surface mucus layer, rather than *Symbiodinium*. Similarly, previous studies also showed superoxide production at the surface of aquaria-hosted pigmented colonies of *P. astreoides* and both bleached and pigmented colonies of *S. pistillata* in the absence of light, pointing to non-algal superoxide sources^18^,^40^. Yet in the presence of light, superoxide production by *S. pistillata* was moderately elevated for pigmented colonies but not bleached corals in this previous study^18^. This result was interpreted to indicate a potential indirect role for algae in stimulating coral-derived extracellular superoxide in the presence of light (for example, stimulation of coral NAD(P)H oxidase activity via light-enhanced algal NAD(P)H production)^18^. However, we did not observe this effect for corals on a natural reef.

Despite their lack of contribution to external superoxide production by corals, *Symbiodinium* cells are still undoubtedly a source of internal superoxide to their coral hosts (see ref. 12 and references therein). Indeed, the ability of cultured *Symbiodinium* isolates to generate extracellular superoxide^18^,^40^ demonstrates that *Symbiodinium*-derived superoxide may have the potential to directly interact with host gastrodermal cells. In contrast to superoxide, external hydrogen peroxide may ultimately derive from *Symbiodinium*. For instance, pigmented colonies of *S. pistillata* were previously found to produce external hydrogen peroxide, yet bleached colonies did not—thereby implicating symbiotic algae as the source^48^. In this case, the transport of internal hydrogen peroxide into seawater may help to maintain redox homoeostasis^48^. Yet non-*Symbiodinium* sources of superoxide at the coral surface are also likely to contribute indirectly to external hydrogen peroxide concentrations, since the dismutation of superoxide produces hydrogen peroxide. Regardless of the ability of *Symbiodinium* to generate ROS within coral tissues, however, recent evidence has demonstrated a lack of correspondence between coral host and *Symbiodinium* redox metabolism during bleaching conditions, suggesting that *Symbiodinium*-derived ROS may not be the ultimate trigger of coral bleaching^53^,^54^.

### Coral microbiome community composition

Many prokaryotic groups associate with diverse species of corals and are thought to benefit their host by recycling nutrients and producing antibiotic compounds^55^. To investigate potential microbial population-level control over external superoxide concentrations, we examined the bacterial and archaeal community composition of corals in Kaneohe Bay, with the exception of *Fungia scutaria*, which was not sampled due to permit restrictions. Sequencing of small subunit ribosomal RNA gene amplicons revealed the presence of common coral bacterial genera, including *Endozoicomonas*, which was particularly abundant in *P. compressa* (on average 56% and 34% of sequences within bleached and healthy *P. compressa* colonies, respectively), and minimal archaeal sequences. In addition, members of *Verrucomicrobia* and *Planctomycetes* were prominently associated with *M. capitata* and *P. damicornis* (Supplementary Table 4). Moreover, like superoxide production, microbial community composition varied significantly as a function of coral species (ANOSIM, *R*=0.79, *P*=0.01) but not health state (pigmented versus bleached) (ANOSIM, *R*=−0.035, *P*=0.62) (Fig. 4l). Pairwise comparisons of the microbial community composition assessed using Bray–Curtis similarity revealed that each species was significantly different from the others (ANOSIM, *R*=0.52–0.99; *P*<0.05), except for *P. damicornis* and *M. capitata* (ANOSIM, *R*=0.56, *P*=0.07), which exhibited the most similar external superoxide levels among the species examined for microbial community composition (Fig. 1b). This community analysis takes into account bacteria associated with the coral mucus and tissue, but probably only the mucus-associated microbes contribute directly to external superoxide levels (see above). Nevertheless, these results are in line with other studies demonstrating that coral microbial communities are species-specific^56^,^57^. Thus, the coral microbiome may contribute to coral species-specific external superoxide production.

### Superoxide from bacteria and asymbiotic coral larvae

To evaluate the coral host and epibiotic bacteria as potential sources of superoxide on the coral surface, we measured extracellular superoxide production by symbiont-free (asymbiotic) coral larvae and coral-derived bacterial symbionts *in vitro*. The majority of coral species examined in Kaneohe Bay vertically inherit *Symbiodinium* from maternal colonies, precluding the ability to collect and interrogate symbiont-free larvae. Therefore, to assess the coral host's ability to produce extracellular superoxide in the absence of *Symbiodinium* or bacterial symbionts, gametes were obtained from broadcast spawning corals on a reef in Curaçao. Following fertilization and rearing in sterile seawater (at least 24 h of development), asymbiotic larvae of the corals *Orbicella faveolata*, *Diploria labyrinthiformis* and *Colpophyllia natans* produced extracellular superoxide at considerably high rates (260–821 fmol larva^−1^ h^−1^) (Supplementary Table 5). Disrupted electron transport in mitochondrial membranes has been suggested as the primary pathway of superoxide production in stressed symbiotic cnidarians^58^,^59^. However, our results indicate a pathway of superoxide production by healthy coral larvae that is independent of intracellular sources because our method only detects extracellular superoxide^17^ and the superoxide anion does not readily cross healthy biological membranes^50^. Although larval responses do not necessarily reflect superoxide dynamics in adult corals, these results nonetheless demonstrate the potential of several coral species to produce extracellular superoxide independently of their microbial symbionts.

In addition to asymbiotic coral larvae, we also examined coral-associated bacteria for their ability to make extracellular superoxide *in vitro*. A wide phylogenetic and ecological diversity of heterotrophic bacteria, including genera commonly found in corals, were recently shown to produce extracellular superoxide^17^. However, bacteria specifically isolated from corals were not examined as part of that study. As such, we confirmed that representative coral bacteria have the ability to produce extracellular superoxide, including an example of the widespread and numerically abundant coral symbiont *Endozoicomonas*^60^ *(E. montiporae*, LMG 24815), which was a prevalent organism in the *Porites* spp. microbiomes studied in Kaneohe Bay. We tested other common coral bacteria, cultured from corals in Micronesia, which also produced substantial extracellular superoxide, including *Ruegeria* sp. (isolate WHOIMSCC16 from *S. pistillata*) and *Vibrio* sp. (isolate WHOIMSCC50, from *P. lobata*). Extracellular superoxide production by these coral-derived bacteria ranged from 0.13±0.06 to 2.2±1.2 amol cell^−1^ h^−1^ (Supplementary Table 5), which is similar to extracellular superoxide production by other heterotrophic bacteria^17^.

## Discussion

Overall, our findings demonstrate that corals and/or their microbial epibionts regulate external superoxide levels in a species-specific manner, which suggests an important role for external superoxide in the physiology and health of the coral holobiont. Most current theoretical models of coral bleaching are based on internal biochemical dynamics, especially the build-up of ROS within coral tissues. Alternatively, external ROS fluxes may be involved in bleaching, as well. For example, addition of the ROS scavengers ascorbate and catalase decreased bleaching in *Agaricia tenuifolia* in a previous study^61^. Although ascorbate is a small molecule that can be transported across cell membranes, catalase is a large enzyme that cannot readily penetrate the cell surface, unless it is actively engulfed via endocytosis. Thus, at least some benefit of these exogenous antioxidants may be explained by a decrease in external rather than internal ROS levels. Indeed, exogenous catalase^61^ or active release of hydrogen peroxide by the coral itself^48^ may alleviate internal redox stress and thereby protect the coral from ROS-induced bleaching.

Elevated ROS levels are commonly assumed to play an antagonistic role in corals (that is, oxidative stress), but ROS may also be beneficial to coral physiology. In fact, healthy corals, *Symbiodinium* cells, and bacteria produce extracellular superoxide and hydrogen peroxide under non-stressful conditions, confirming that ROS production is not always correlated to oxidative stress^17^,^18^,^40^,^48^. Similarly, a previous study documented basal levels of singlet oxygen in the symbiotic anemone *Aiptasia*, which did not increase during heat-induced bleaching under low illumination^62^. In a wide range of macro- and microorganisms, extracellular superoxide production is a beneficial trait commonly mediated by transmembrane or outer membrane oxidoreductases (for example, NAD(P)H oxidases, peroxidases), as seen in model bacteria, non-symbiotic phytoplankton and the symbiotic anemone *Nematostella vectensis*^63^,^64^,^65^,^66^. For example, oxidoreductase-mediated production of extracellular superoxide promotes cell division and differentiation in a variety of organisms, including fungi^24^, microbial eukaryotes^24^ and bacteria^22^,^23^. Enzymatic production of extracellular superoxide is also involved in wound repair by plants^66^, defence against epiphytic parasites in macroalgae^28^ and the immune response of mammalian leucocytes^27^. Interestingly, in a previous study, the oxidoreducatase inhibitor diphenylene iodinium impeded extracellular superoxide production by a *Symbiodinium* clade C representative (CCMP 2466) as well as bleached and pigmented colonies of *S. pistillata*^18^. These results suggest that oxidoreductases are involved in extracellular superoxide production by corals and their symbionts and, moreover, these findings underscore the potentially beneficial roles of extracellular superoxide in coral physiology and health^24^,^63^.

The potential beneficial roles of extracellular ROS in coral function and health are likely diverse and vary in the presence and absence of external stressors. For example, according to a recent study, corals may release external hydrogen peroxide to facilitate feeding on zooplankton or as a mode of defence against pathogens regulated by physical and chemical stimuli^67^. Consistent with the ability of superoxide to act as a cell density-dependent growth promoter, and with previous observations from phytoplankton and heterotrophic bacteria^68^, we found that extracellular superoxide production by actively developing coral larvae was inversely related to larval density (Supplementary Fig. 3). Furthermore, several previous studies suggest a possible role of NAD(P)H oxidoreductases^19^,^20^ and extracellular superoxide production^21^ in the coral immune defence system. Recent evidence even indicates that bacteria associated with the model organism *Caenorhabditis elegans* protect their host from parasites by generating superoxide^29^, suggesting that a similar mutualism may be present in corals. Although our results show that external superoxide production is decoupled from bleaching and symbiont abundance within a single coral species, extracellular superoxide production may be inversely related to bleaching susceptibility across coral species. For example, *Porites* spp., which produced the highest external superoxide concentrations, are among the most resilient species to thermal bleaching, while *Montipora* spp., which had the lowest superoxide levels, are more susceptible^69^. While purely speculative at this point, this potentially beneficial role of external superoxide contradicts previous observations that exogenous antioxidants protect against bleaching^61^, highlighting the need to further investigate the possible beneficial versus detrimental roles of external superoxide in coral immune defence, development and overall health.

Given the light-independent and species-specific control of superoxide levels revealed here, coupled with similar findings for aquaria-hosted corals grown under non-stressful conditions (refs 18, 40), it is clear that external superoxide plays a role in coral physiology that may also be species-specific. Although the role of external superoxide in coral biology and health remains unclear, it may be involved in a range of positive and negative functions, from pathogen defence to bleaching. On the basis of the possible species-specific health effects of external superoxide, whether beneficial or detrimental, we speculate that the capacity of corals to regulate superoxide levels in their immediate vicinity may potentially underlie the ecological distribution of species and/or species-level bleaching patterns on reefs. Indeed, preliminary comparison of the species-specific superoxide levels that we observed to broad trends in interspecific bleaching patterns^69^ suggests that external superoxide may be inversely related to bleaching susceptibility. Clearly, targeted investigations of this correlation, as well as other links between superoxide and coral health and function, should follow. Although superoxide is an important target for future study (for example, it is directly linked to the apoptosis pathway^15^ involved in coral bleaching^16^ and yet facilitates essential physiological functions), the internal and external roles of other ROS such as hydrogen peroxide and hydroxyl radical should also be more fully examined and incorporated into coral physiological models. Additional direct measurements of ROS production and degradation by the coral holobiont and diverse individual members of this community will advance a more holistic view of ROS cycling within corals. In turn, these advancements will improve our current understanding of coral ecosystem health and development, and ultimately, the future of coral reefs under sustained climate change.

## Methods

### Reef sites, corals and sampling

*In situ* superoxide measurements were conducted between 10:00 and 15:00 hours on pigmented and bleached colonies of *Porites compressa*, *Porites lobata*, *Montipora capitata*, *Pocillopora damicornis* and *Fungia scutaria*, at six different reef sites in Kaneohe Bay, Hawaii including sites A (21.4599° N, 157.8228° W), B (21.45502° N, 157.8226° W), C (21.46073° N, 157.8225° W), D (21.45443° N, 157.8034° W), E (21.46135° N, 157.793° W) and F (21.45702° N, 157.8002° W) (Supplementary Fig. 1). Small tissue and skeletal pieces were removed from all colonies except *F. scutaria* (due to lack of a permit) under the State of Hawaii Department of Land and Natural Resources Special Activity Permit #2015-49 using a hammer and chisel, placed on ice for no more than 3 h, and frozen to −80 °C. Additionally, diel superoxide measurements were conducted on adjacent colonies of *Porites compressa* located off the Point of Coconut Island (Site P), (21.43286° N, 157.7863° W). We were not permitted to remove tissue from colonies at this site. For diel measurements, photosynthetically active radiation (PAR) was measured using an underwater sensor (LI-COR, data collected by a LI-1500 light sensor logger).

### *In situ* superoxide measurements

Superoxide concentrations were measured with a flow-through FeLume Mini system (Waterville Analytical, Waterville ME) via the specific reaction between superoxide and the chemiluminescent probe methyl *Cypridina* luciferin analogue (MCLA, Santa Cruz Biotechnology). The FeLume system is composed of two separate fluid lines, one of which is dedicated to the analyte solution and the other to the MCLA reagent. The reagent and, as indicated, the analyte solutions, are amended with 50 μM diethylene-triaminepentaacetic acid (DTPA) to sequester trace metal contaminants that would otherwise significantly reduce the lifetime of superoxide. To measure superoxide, both the analyte solution and the MCLA reagent are independently flushed through the FeLume system at an identical flow rate using a peristaltic pump. The MCLA reagent consisted of 4.0 μM MCLA (similar to concentrations used previously and by other investigators^38^,^39^,^70^,^71^) in 0.10 M MES with 50 μM DTPA adjusted to pH 6.0. The solutions converge in a spiral flow cell immediately adjacent to a photomultiplier tube, which continuously acquires data that is displayed in real time using a PC interface. Similar systems have been used to generate high sensitivity measurements of natural superoxide concentrations and decay rates^36^,^70^, as well as extracellular superoxide production by bacteria^17^ and phytoplankton isolates^37^,^72^.

For *in situ* superoxide measurements, the FeLume was hosted onboard a small boat. To eliminate abiotic photochemical processes that may produce superoxide during *in situ* measurements, opaque tubing was used, and the entire analytical system was shielded from light. The MCLA reagent was kept on ice during all field analyses, which greatly improved the stability of measurements. Seawater was directly pumped into the FeLume by placing the analyte tubing at discrete positions millimetres above the coral surface at specific points of interest, as indicated in the main text and figure legends, or in the surrounding seawater with the help of a snorkeller. To minimize the length of tubing (travel time) required to transport the water from the coral surface to the instrument, the boat was positioned adjacent to the coral being measured (but without shading the coral). Flow rates were consistent within FeLume runs and varied from 6 to 8 ml min^−1^ between runs.

For *in situ* superoxide measurements on the reef, we first ran a reagent blank on the boat to account for superoxide generated from the autooxidation of MCLA^36^. The reagent blank consisted of aged filtered reef water (AFRW), which was collected the previous day from the same depth as the corals (<0.5 m), filtered gently (0.2 μm Sterivex, Millipore), aged in the dark overnight (>12 h) and supplemented with DTPA (50 μM), and then aged for an additional >12 h. The AFRW+DTPA solution was kept at *in situ* temperature by suspending it in a bottle in the water alongside of the boat. By doing this, slight increases in the baseline signal with increasing *in situ* temperature as observed previously^36^ were eliminated. Signal from the reagent blank was acquired for ∼2 min to generate stable chemiluminescent baseline signals (<4% coefficient of variation). The tubing was then passed to a snorkeller, who held the tubing at the seawater surface, ∼5–15 cm from the coral surface, at the coral surface (millimetre scale, without touching the coral), and then back to seawater background positions. The tubing was slowly moved along the coral surface to minimize entrainment of background seawater. Signals were collected until a relatively stable, steady-state reading was achieved and maintained for at least 2 min. Finally, SOD was added (0.8 U ml^−1^, final) to an aliquot of water taken from the coral surface to confirm that superoxide was responsible for the signal observed. SOD routinely lowers seawater baseline signals, reflecting the non-zero concentration of superoxide in the seawater (seawater blank), as well as the reagent blank. The coral-derived signal was obtained by subtracting the seawater signal obtained at 5–15 cm away from the coral surface from the coral surface signal, which removes both the seawater blank and reagent blank. Coral-derived superoxide concentrations were not corrected for superoxide decay during sampling and thus represent conservative estimates. The surface and coral-depth background seawater signals were corrected by subtracting the AFRW+DTPA baseline (that is, subtraction of the reagent blank only).

Corrected signals were converted to superoxide concentrations via calibration with multi-point standard curves using the superoxide source potassium dioxide (KO_2_). The calibrations were conducted in the lab using the same tubing, flow rate and temperature as the *in situ* measurements. Considering the short lifetime of superoxide, standards were prepared immediately before analysis. Primary stock solutions were made by dissolving a small quantity of KO_2_ in a basic matrix (0.03 N NaOH, 50 μM DTPA, pH=12.5). Superoxide concentrations in primary standards were quantified by measuring the difference in absorbance at 240 nm before and after the addition of SOD (∼8 U ml^−1^, final) and then converting to molar units based on the molar absorptivity of superoxide (2,183 l mol^−1^ cm^−1^ at 240 nm, pH=12.5, and corrected for the absorption of hydrogen peroxide formed during decay)^73^. Primary stocks had to be substantially diluted to generate representative concentrations for analysis on the FeLume. To generate secondary stocks, the primary stock solution was diluted with AFRW+DTPA. Final superoxide concentrations in secondary stocks were 3–38 nM.

For each calibration point, a separate FeLume run was conducted as follows: first, a blank AFRW+DTPA solution was allowed to react with the MCLA reagent until a stable baseline (<4% coefficient of variation) was achieved for ∼1 min. Then the secondary standards were pumped directly into the FeLume, and the decay of superoxide was monitored for at least 1 min. Finally, SOD was added to the secondary standard (∼0.8 U ml^−1^, final) to confirm that the signal was attributable to superoxide. In all cases, the chemiluminescence signal decreased rapidly to or slightly below AFRW+DTPA baseline levels after the addition of SOD. Chemiluminescence signals collected during the decay of superoxide were extrapolated backwards in time (0.28–63 s) to the point when the primary standard was quantified. Extrapolations assumed first-order decay kinetics because decay data were log-linear.

Calibration curves were constructed on the basis of the linear regression of multiple standard points (extrapolated luminescence versus superoxide concentration). Calibrations yielded linear curves (for example, *R*>0.93), with a sensitivity that ranged from 0.1 to 0.4 luminescence units per pM superoxide. The half-life of superoxide in the calibration matrices ranged from 0.41 to 0.56 min. These half-lives represent maximum estimates of the lifetime of superoxide on the reef because they were derived from AFRW amended with DTPA, which sequesters trace metals that would otherwise significantly reduce the lifetime of superoxide. The detection limit of this method, calculated as three times the s.d. of a series of blank measurements, was 0.24 nM. Given the sensitivity of superoxide to trace metal contamination, all vials and glassware were pre-cleaned with 10% HCl and washed with ultrapure (18 MΩ) water before use. All reagents were trace metal grade.

### *Symbiodinium* cell counts from coral tissue

Frozen coral tissues were defrosted on ice and tissue was removed from the skeleton using an airbrush with 0.2 μm filtered seawater, blended for 30 s and centrifuged at 5,000 r.p.m. for 5 min. The supernatant was discarded, and the algal pellet was resuspended in filtered seawater and repeatedly vortexed and pelleted until the symbiont cells were free of host material. The algal pellet was finally preserved in 4% paraformaldehyde with filtered seawater and stored at 4 °C. For quantification, cells were concentrated onto 5 μm membrane filters (Millipore), mounted onto slides and imaged using Cy5 and TL DIC channels simultaneously with × 20 objective and × 1.6 optovar with a Zeiss Axio Observer.Z1 microscope and AxioCam MRm Rev.3w camera (Carl Zeiss Inc). Each image contained 1,388 × 1,040 pixels, with each pixel sized 0.32 × 0.32 μm, relevant to a 447.63 × 335.40 μm area. For each sample, 12 images from 12 fields of view under the same imaging conditions were captured with Zen 2011 software (Carl Zeiss, Inc.), and cell counts were then automated using a custom Matlab script and normalized to skeletal surface area, which was obtained from either a cork borer diameter (for *Porites*) or using aluminium foil (other colonies)^74^. Replicate *Symbiodinium* counts on the same specimen typically agreed within ±5% (s.d.).

### Coral microbiome community analysis

Samples of coral tissue were collected after the superoxide measurements using a hammer and chisel and were placed into whirl-pack bags underwater. Samples were kept on ice until arriving back at the laboratory, where they were wrapped in aluminium foil and frozen to −80 °C until processing. Coral mucus and tissue were airbrushed from the coral tissue as previously described^75^, and DNA was extracted using the UltraClean Tissue and Cells DNA isolation Kit (Mo Bio Laboratories) with the addition of Proteinase K digestion (15 μl; 20 mg ml^−1^ at 60 °C for 30 min). DNA was quantified with the Qubit HS dsDNA fluorescent assay (Invitrogen), and samples were shipped to the University of Illinois for amplification and sequencing. A mastermix for each sample was prepared using the Roche High Fidelity Fast Start Kit and 20 × Access Array loading reagent. Mastermix was aliquoted to 48 wells of a PCR plate. To each well, 1 μl DNA and 1 μl Fluidigm Illumina linkers with unique barcode were added. Primer sequences for respective variable regions^76^ with Fuidigm CS1 (for 515F) and CS2 5′ (for 806R) tails (non-underlined) included: V4-515F: 5′-ACACTGACGACATGGTTCTACAGTGYCAGCMGCCGCGGTAA-3′ and V4-806RB: 5′-TACGGTAGCAGAGACTTGGTCTGGACTACNVGGGTWTCTAAT-3′ primers^76^. A 4 μl sample aliquot was loaded in the sample inlets and 4 μl of primer loaded in primer inlets of a previously primed Fluidigm 48.48 Access Array integrated fluidic circuit (IFC). The IFC was placed in an AX controller (Fluidigm Corp.) for microfluidic loading of all primer/sample combinations. Following the loading stage, the IFC plate was loaded on the Fluidigm Biomark HD PCR machine and samples were amplified using the default Access Array cycling programme without imaging. Following amplification, 2 μl of Fluidigm Harvest Buffer was loaded in the sample inlets and loaded on the AX controller for harvesting PCR products. Collected product was then transferred to a new 96 well plate quantified on a Qubit fluorometer and stored at −20°C. All samples were run on a Fragment Analyzer (Advanced Analytics, Ames, IA) and amplicon regions quantified. Samples were then pooled in equal amounts according to product concentration. The pooled products were then size selected on a 2% agarose E-gel (Life Technologies) and extracted from the isolated gel slice with a gel extraction kit (Qiagen). Cleaned size-selected product was run on an Agilent Bioanalyzer to confirm appropriate profile and determination of average size. The final pooled Fluidigm library pool was quantitated by qPCR on a BioRad CFX Connect Real-Time System (Bio-Rad Laboratories, Inc. CA) to ensure accuracy of quantitation of the library containing properly adapted fragments. The final denatured library pool was spiked with 15% non-indexed PhiX control library provided by Illumina and loaded onto the MiSeq V2 flow cell at a concentration of 8 pM for cluster formation and sequencing (Illumina). The PhiX control library provides a balanced genome for calculation of matrix, phasing and pre-phasing, which are essential for accurate basecalling. The libraries were sequenced from both ends of the molecules to a total read length of 250 nt from each end. Sequence analyses were conducted using mothur v.3.3.3 (ref. 77) and included assembly of the paired ends, amplicon size selection and alignment to the SSU rRNA gene. Chimeras were detected using UCHIME^78^ and subsequently removed. Taxonomic classification of sequences was conducted with the SILVA SSU Ref database (release 123)^79^ using the k-nearest neighbour algorithm, and subsequently chloroplast, mitochrondia and eukaryotic sequences were removed. Sequences were grouped into operational taxonomic units using minimum entropy decomposition^80^ for statistical analysis using Primer-E (v.7.0.9, Primer- E Ltd.).

### Cultivation of bacteria

*Endozoicomonas montiporae* (LMG 24815) was obtained from the Belgian Coordinated Collections of Microorganisms. *E. montiporae* was grown in liquid marine broth at 23 °C. *Ruegeria* s.p. (WHOIMSCC16) was obtained from *Stylophora pistillata* and *Vibrio* s.p. (WHOIMSCC50) from *Porites lobata*, both isolated in the Federated States of Micronesia, and were grown using dilute nutrient media (0.8 g nutrient broth, 0.5 g casamino acids, 0.1 g yeast extract, 860 ml seawater and 140 ml sterile water). Growth of all bacteria was quantified with cell counts after staining with 2-(4-amidinophenyl)-1H-indole-6-carboxamidine (DAPI).

### Collection and rearing of coral larvae

Egg and sperm from at least three colonies of *Orbicella faveolata*, *Colpophyllia natans* and *Diploria labyrinthiformis* were collected in the evening 6–12 days following the full moon at the Water Factory site in Curaçao (12.11298° N, 68.96103° W) using standard collection nets. The gametes were pooled and fertilized for ∼30 min, initially diluted into 0.45 μm filtered seawater and subsequently maintained in 0.2 μm filtered seawater with daily water changes once they developed into larvae.

### Extracellular superoxide measurements of cultures and larvae

Extracellular superoxide produced by laboratory cultures and coral larvae was measured using a previously described MCLA/FeLume method^17^ with a few modifications (see above for general description of the FeLume system). Briefly, carrier solutions were gently pumped (2 ml min^−1^) across a sterile syringe filter placed in the FeLume's analyte line for ∼2 min to generate stable baseline signals (<4% coefficient of variation). For larvae, the carrier solution consisted of AFRW amended with 75 μM DTPA (AFRW+DTPA). For bacteria, the carrier solution was 20 mM phosphate-buffered (pH=7.6) artificial seawater (NaCl, 0.3 M; MgSO_4_, 50 mM; CaCl_2_, 10 mM; KCl, 10 mM) amended with 75 μM DTPA (PBASW+DTPA). Next, the pump was stopped, the syringe filter was removed and using a syringe, specimens were gently deposited on the filter (larvae=10 μm, bacteria=0.2 μm). Then the specimen-loaded filter was placed back inline, and the pump was restarted (2 ml min^−1^). In principle, superoxide produced extracellularly is entrained by the carrier solution and detected upon mixing with MCLA in the flow cell downstream of the specimens. Biological signals were collected until a stable, steady-state reading was achieved (<∼4% coefficient of variation) and maintained for at least 1 min. Finally, SOD was added to the carrier solution (0.8 U ml^−1^, final) to confirm that superoxide was responsible for the signal observed.

Stable biological signals were averaged and corrected for background luminescence by subtracting the average initial baseline (that is, obtained with the clean syringe filter inline, immediately before the addition of organisms and without the addition of SOD). Corrected biological signals were converted to superoxide concentrations via calibration with multi-point KO_2_ standard curves under identical conditions as biological experiments, similarly to the protocol described elsewhere^17^ and outlined above for *in situ* superoxide measurements. Under the conditions used for these experiments (for example, flow rate, flushing volume, specimen load and so on), steady-state superoxide concentrations ranged from 0.22 to 12 nM. The typical detection limit (defined as three times the average s.d. of replicate baseline signals) was 0.24±0.11 nM (avg±s.d.). As determined from calibration experiments, superoxide half-lives varied inversely with superoxide concentration and ranged from 0.7 to 3.3 min (AFRW+DTPA in Curaçao) and 4.9 to 24.8 min (PBASW+DTPA). Some half-lives in PBASW+DTPA are relatively high compared with most natural waters, but similar to superoxide decay rates measured in water samples from the Costa Rica Dome that were also filtered and amended with DTPA^37^. Moreover, the relatively long half-lives we measured in PBASW+DTPA are not surprising because, unlike natural waters, PBASW lacks any organics that could contribute to the decay of superoxide. Thus, these half-lives do not and are not meant to accurately represent the lifetime of superoxide in the presence of corals, their symbionts or natural seawater.

Extrapolation times were typically 64.6±10.6 s (avg±s.d.) under our experimental conditions. Biogenic superoxide concentrations were not corrected for superoxide decay and thus represent conservative estimates. As above, calibrations yielded highly linear curves (for example, *R*>0.97 for Curaçao AFRW+DTPA; *R*>0.98 for PBASW+DTPA), with a sensitivity that averaged 2.2±0.6 pM per luminescence count. Net superoxide production rates were calculated as the product of the steady-state superoxide concentration and flow rate (final units of amol per hour). The production rate of superoxide by each replicate was normalized to the total number of cells or larvae loaded on the filter (final units of amol per organism per hour).

### Statistical analysis

Superoxide data and *Symbiodinium* counts were analysed using JMP Pro 12.1.0 statistical analysis software. The effects of coral species and health state on average seawater-normalized superoxide levels (Fig. 1b) were examined via full factorial ANOVA and *post-hoc* analysis via Tukey's honest significant difference test. Mixed-factor repeated measures ANOVA was used to evaluate the effects of PAR and health state on time-series superoxide measurements from *Porites compressa* (Fig. 2). Species-specific *Symbiodinium* counts for bleached versus pigmented colonies (Fig. 4k) were performed using two-sample *t*-tests assuming unequal variance. Other two-sample comparisons (for example, bleached versus pigmented superoxide levels for a single species (Fig. 1b), superoxide levels between species (Fig. 3b) and superoxide levels between corals and bulk seawater (Fig. 3b) were also assessed with Student's *t*-test. Statistical analysis of microbiome sequence data was conducted using Primer (v.7, Primer-E Ltd, Ivybridge, UK).

### Data availability

Full-length SSU rRNA gene sequences of the coral bacterial cultures are available in GenBank under accession numbers KT957318 and KT957319. Amplicon sequence data are available in as NCBI bioproject PRJNA324813. Additional data may be available from the authors upon request.

## Additional information

**How to cite this article:** Diaz, J. M. *et al*. Species-specific control of external superoxide levels by the coral holobiont during a natural bleaching event. *Nat. Commun.* **7**, 13801 doi: 10.1038/ncomms13801 (2016).

**Publisher's note**: Springer Nature remains neutral with regard to jurisdictional claims in published maps and institutional affiliations.

## Supplementary Material

Supplementary Information Supplementary Figures 1-3 and Supplementary Tables 1-5
