# Supplementary material for: Species-specific control of external superoxide levels by the coral holobiont during a natural bleaching event
Source: Nat Commun. 2016 Dec 7;7:13801. doi: 10.1038/ncomms13801 (PMC5150980; doi:10.1038/ncomms13801)
Supplement: Supplementary Information — Supplementary Figures 1-3 and Supplementary Tables 1-5 [file ncomms13801-s1.pdf]

**Supplementary Figures**

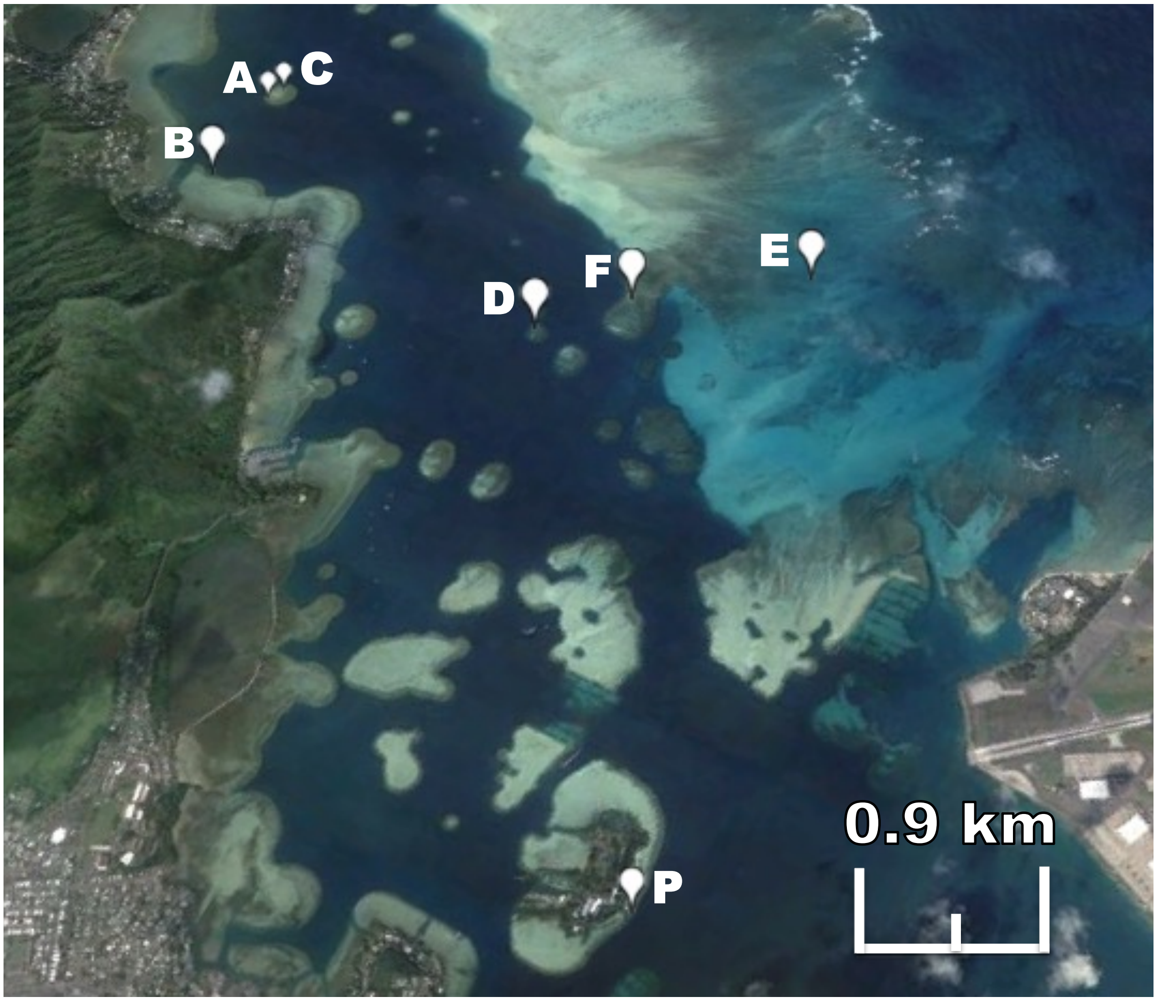


**Supplementary Figure 1. Field sites.** Coral-derived superoxide was measured in Kaneohe Bay, Hawaii at sites A (21.4599° N, 157.8228° W), B (21.45502° N, 157.8226° W), C (21.46073° N, 157.8225° W), D (21.45443° N, 157.8034° W), E (21.46135° N, 157.793° W), F (21.45702° N, 157.8002° W), and P (21.43286° N, 157.7863° W). Map data: Google, DigitalGlobe.

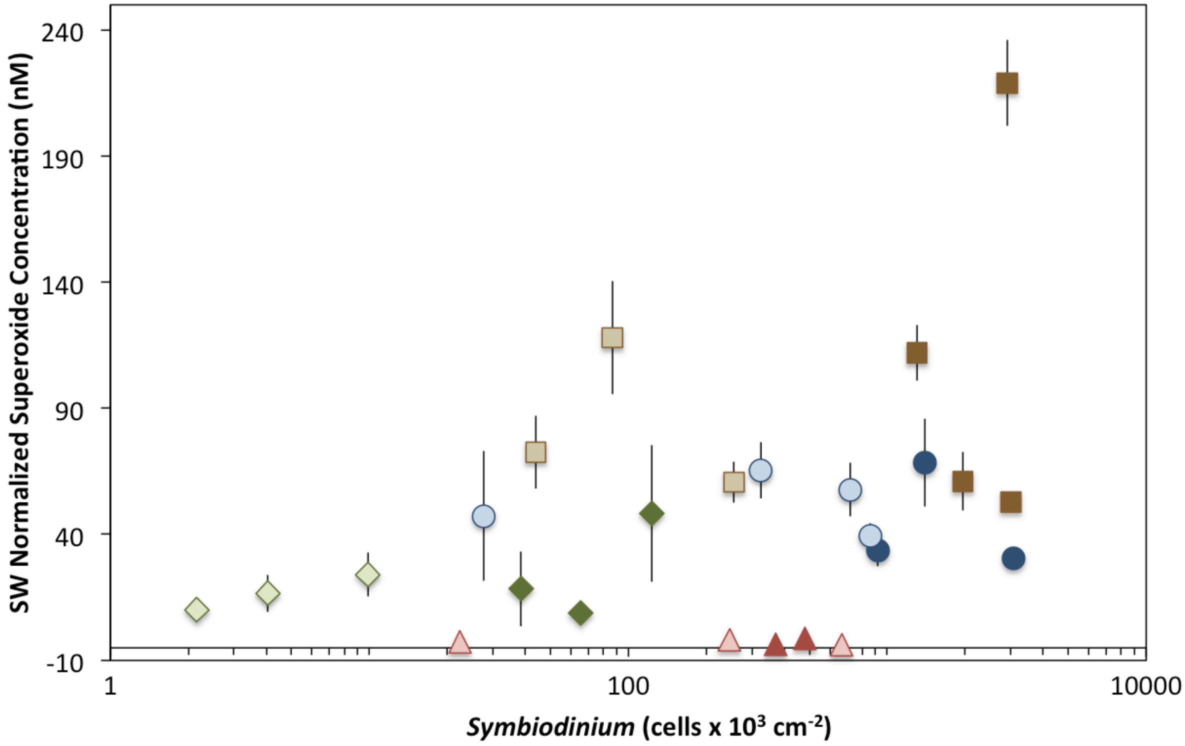


**Supplementary Figure 2. Lack of significant correlation between coral-derived superoxide levels and the abundance of *Symbiodinium* cells in coral tissue.** Bleached (lighter color) and pigmented (darker color) specimens of *M. capitata* (red triangles), *P. damicornis* (green diamonds), *P. compressa* (blue circles), and *P. lobata* (brown squares) are represented. Pearson correlation coefficients for *M. capitata*, *P. damicornis*, *P. compressa*, and *P. lobata* datasets (based on at least five data points) were -0.28 (p= 0.65), 0.71 (p=0.11), -0.44 (p=0.33), and 0.31 (p=0.50), respectively. Error bars for SW normalized superoxide represent SD (n=8-16). Replicate *Symbiodinium* counts on the same specimen typically agreed within ± 5% (SD).

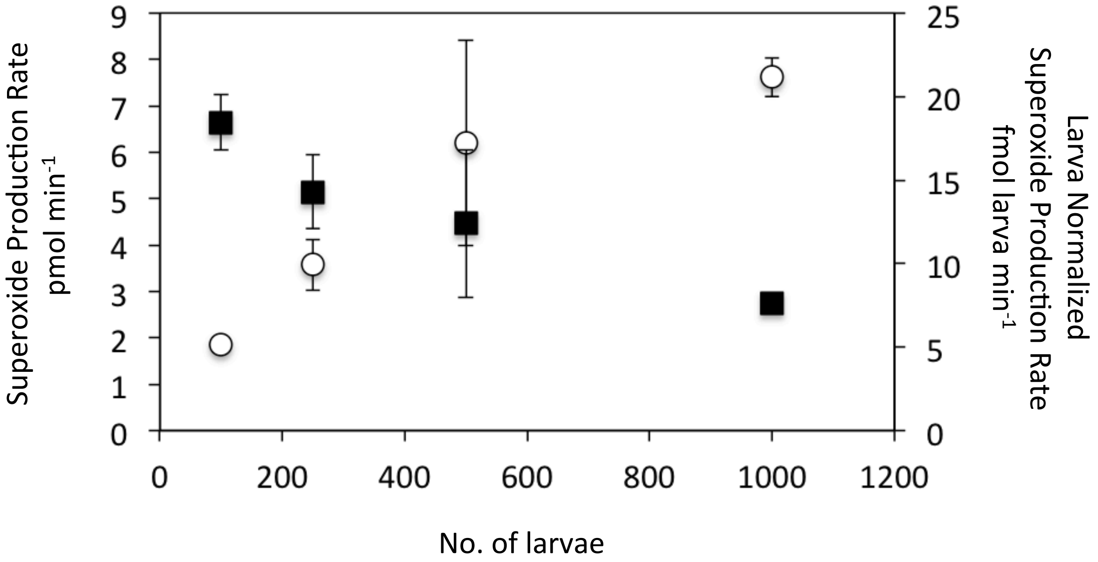


**Supplementary Figure 3. Total (white circles) and larva-normalized (black squares) extracellular superoxide production rates by *D. labyrinthiformis* larvae.** Production rates were calculated based on steady-state superoxide levels generated by larvae housed on a syringe filter in-line with the FeLume system. Error bars represent the range of measurements for two biological replicates.

## Supplementary Tables

**Supplementary Table 1. Superoxide data from all coral species.** Superoxide concentrations in background seawater (which were 4 – 11 nM) have been subtracted from coral-derived superoxide concentrations. Superoxide at the surface of *M. capitata* was routinely lower than background seawater, resulting in negative seawater-normalized concentrations.

| | | | | | | Corrected $[O_2^-]$ (nM) | | | Signal | Corrected $[O_2^-]$ (nM) | |
|---|---|---|---|---|---|---|---|---|---|---|---|
| Site | File Name | Coral No. | Coral Species | Health | Spot | Avg signal | SD | Peak | Duraction (s) | Coral Avg | SD |
| A | kba_2 | 3 | *M capitata* | bleached | 1 | -2.86 | 0.73 | -0.61 | 36 | -1.79 | 0.87 |
| A | kba_2 | | *M capitata* | bleached | 2 | -1.09 | 1.13 | 1.90 | 36 | | |
| A | kba_2 | | *M capitata* | bleached | 3 | -1.08 | 2.29 | 3.58 | 34 | | |
| A | kba_2 | | *M capitata* | bleached | 4 | -2.13 | 0.63 | -0.61 | 32 | | |
| A | kba_2 | 4 | *M capitata* | pigmented | 1 | -1.72 | 1.00 | 0.22 | 16 | -1.38 | 0.48 |
| A | kba_2 | | *M capitata* | pigmented | 2 | -1.04 | 0.98 | 1.27 | 36 | | |
| A | kba_2 | 5 | *P compressa* | bleached | 1 | 50.68 | 4.15 | 55.03 | 10 | 57.67 | 10.59 |
| A | kba_2 | | *P compressa* | bleached | 2 | 52.47 | 4.77 | 59.86 | 18 | | |
| A | kba_2 | | *P compressa* | bleached | 3 | 69.85 | 4.71 | 79.03 | 26 | | |
| A | kba_2 | 6 | *P compressa* | pigmented | 1 | 33.44 | 2.43 | 37.53 | 15 | 30.5 | 4.16 |
| A | kba_2 | | *P compressa* | pigmented | 2 | 27.55 | 2.99 | 31.45 | 21 | | |
| B | kbb_1 | 9 | *P compressa* | pale | 1 | 40.78 | 3.70 | 46.78 | 18 | 39.46 | 4.99 |
| B | kbb_1 | | *P compressa* | pale | 2 | 43.66 | 5.34 | 55.90 | 21 | | |
| B | kbb_1 | | *P compressa* | pale | 3 | 33.95 | 4.20 | 41.65 | 16 | | |
| B | kbb_1 | 7 | *P compressa* | bleached | 1 | 25.32 | 0.87 | 24.78 | 10 | 47.31 | 25.87 |
| B | kbb_1 | | *P compressa* | bleached | 2 | 40.79 | 3.54 | 44.58 | 10 | | |
| B | kbb_1 | | *P compressa* | bleached | 3 | 75.81 | 6.14 | 83.78 | 10 | | |
| B | kbb_1 | 8 | *P compressa* | pigmented | 1 | 38.28 | 2.69 | 42.59 | 24 | 33.78 | 6.36 |
| B | kbb_1 | | *P compressa* | pigmented | 2 | 29.28 | 2.65 | 33.05 | 18 | | |
| B | kbb_2 | 12 | *M capitata* | bleached | 1 | -2.37 | 0.70 | -1.10 | 28 | -2.65 | 0.32 |
| B | kbb_2 | | *M capitata* | bleached | 2 | -2.99 | 0.77 | -0.48 | 38 | | |
| B | kbb_2 | | *M capitata* | bleached | 3 | -2.58 | 1.04 | 0.46 | 31 | | |
| B | kbb_2 | 11 | *M capitata* | pigmented | 1 | -2.69 | 0.66 | -0.58 | 43 | -3.64 | 0.85 |
| B | kbb_2 | | *M capitata* | pigmented | 2 | -4.31 | 0.48 | -3.41 | 45 | | |
| B | kbb_2 | | *M capitata* | pigmented | 3 | -3.91 | 0.51 | -2.99 | 37 | | |
| B | kbb_2 | 10 | *M capitata* | very pale | 1 | -3.54 | 0.45 | -2.68 | 31 | -3.75 | 0.39 |
| B | kbb_2 | | *M capitata* | very pale | 2 | -4.21 | 0.58 | -3.09 | 24 | | |
| B | kbb_2 | | *M capitata* | very pale | 3 | -3.51 | 0.91 | 0.15 | 36 | | |
| C | kbc_1 | 13 | *P damicornis* | bleached | 1 | 8.36 | 0.97 | 10.32 | 13 | 10.03 | 3.35 |
| C | kbc_1 | | *P damicornis* | bleached | 2 | 13.89 | 2.57 | 18.24 | 22 | | |
| C | kbc_1 | | *P damicornis* | bleached | 3 | 7.83 | 1.02 | 10.09 | 16 | | |
| C | kbc_1 | 13 | *P damicornis* | pigmented | 1 | 10.02 | 2.02 | 12.89 | 19 | 8.72 | 1.83 |
| C | kbc_1 | | *P damicornis* | pigmented | 2 | 7.43 | 0.95 | 9.51 | 14 | | |
| C | kbc_3 | 17 | *P damicornis* | pigmented | 1 | 33.47 | 9.06 | 45.75 | 10 | 48.26 | 27.16 |
| C | kbc_3 | | *P damicornis* | pigmented | 2 | 31.70 | 10.41 | 49.59 | 9 | | |
| C | kbc_3 | | *P damicornis* | pigmented | 3 | 79.61 | 17.78 | 109.13 | 16 | | |
| C | kbc_3 | 17 | *P damicornis* | bleached | 1 | 17.77 | 1.58 | 19.77 | 7 | 23.95 | 8.74 |
| C | kbc_3 | | *P damicornis* | bleached | 2 | 30.13 | 3.97 | 34.68 | 11 | | |
| C | kbc_3 | n/a | *F scutaria* | pigmented | 1 | -0.32 | 1.10 | 1.95 | 22 | 2.52 | 4.01 |
| C | kbc_3 | | *F scutaria* | pigmented | 2 | 5.35 | 1.42 | 9.28 | 27 | | |
| C | kbc_3 | n/a | *F scutaria* | bleached | 1 | -0.34 | 0.90 | 2.06 | 30 | 0.76 | 1.55 |
| C | kbc_3 | | *F scutaria* | bleached | 2 | 1.85 | 1.55 | 4.04 | 15 | | |
| C | kbc_4 | 18 | *P damicornis* | bleached | 1 | 7.41 | 0.75 | 9.19 | 15 | 16.59 | 7.34 |
| C | kbc_4 | | *P damicornis* | bleached | 2 | 14.07 | 1.41 | 16.30 | 9 | | |
| C | kbc_4 | | *P damicornis* | bleached | 3 | 21.27 | 1.15 | 23.64 | 13 | | |
| C | kbc_4 | | *P damicornis* | bleached | 4 | 23.59 | 1.07 | 24.92 | 7 | | |

## Supplementary Table 1. (continued)

| Site | File Name | Coral No. | Coral Species | Health | Spot | Corrected [$O_2^-$] (nM) Avg signal | SD | Peak | Signal Duraction (s) | Corrected [$O_2^-$] (nM) Coral Avg | SD |
|---|---|---|---|---|---|---|---|---|---|---|---|
| D | kbd_2 | 21 | *P compressa* | pigmented | 1 | 47.59 | 2.29 | 49.97 | 8 | 68.51 | 17.47 |
| D | kbd_2 | | *P compressa* | pigmented | 2 | 63.60 | 5.57 | 72.57 | 16 | | |
| D | kbd_2 | | *P compressa* | pigmented | 3 | 89.15 | 13.32 | 111.71 | 9 | | |
| D | kbd_2 | | *P compressa* | pigmented | 4 | 73.71 | 5.89 | 84.45 | 10 | | |
| D | kbd_4 | n/a | *M capitata* | pigmented | 1 | 0.12 | 0.51 | 1.03 | 27 | -0.52 | 0.57 |
| D | kbd_4 | | *M capitata* | pigmented | 2 | -0.78 | 0.35 | -0.33 | 24 | | |
| D | kbd_4 | | *M capitata* | pigmented | 3 | -0.92 | 0.26 | -0.65 | 22 | | |
| D | kbd_4 | n/a | *P compressa* | pigmented | 1 | 42.72 | 2.51 | 47.16 | 10 | 54.75 | 11.83 |
| D | kbd_4 | | *P compressa* | pigmented | 2 | 55.16 | 3.74 | 59.63 | 16 | | |
| D | kbd_4 | | *P compressa* | pigmented | 3 | 66.36 | 15.59 | 97.26 | 12 | | |
| D | kbd_5 | 25 | *P compressa* | bleached | 1 | 69.54 | 6.16 | 80.01 | 12 | 65.38 | 11.09 |
| D | kbd_5 | | *P compressa* | bleached | 2 | 74.46 | 3.99 | 79.78 | 10 | | |
| D | kbd_5 | | *P compressa* | bleached | 3 | 49.23 | 4.01 | 57.18 | 10 | | |
| D | kbd_5 | | *P compressa* | bleached | 4 | 68.30 | 7.59 | 83.28 | 14 | | |
| E | kbe_1 | 26 | *P lobata* | bleached | 1 | 87.39 | 1.94 | 85.77 | 11 | 72.61 | 14.43 |
| E | kbe_1 | | *P lobata* | bleached | 2 | 58.57 | 1.52 | 64.79 | 10 | | |
| E | kbe_1 | | *P lobata* | bleached | 3 | 71.88 | 0.52 | 73.88 | 10 | | |
| E | kbe_1 | 26 | *P lobata* | pigmented | 1 | 107.72 | 2.76 | 122.46 | 8 | 111.87 | 10.98 |
| E | kbe_1 | | *P lobata* | pigmented | 2 | 124.32 | 5.66 | 153.21 | 9 | | |
| E | kbe_1 | | *P lobata* | pigmented | 3 | 103.56 | 1.90 | 111.27 | 17 | | |
| E | kbe_2 | n/a | *P lobata* | bleached | 1 | 90.74 | 12.56 | 107.28 | 11 | 101.45 | 16.44 |
| E | kbe_2 | | *P lobata* | bleached | 2 | 93.23 | 11.80 | 112.86 | 9 | | |
| E | kbe_2 | | *P lobata* | bleached | 3 | 120.38 | 12.05 | 137.34 | 8 | | |
| E | kbe_2 | n/a | *P lobata* | pigmented | 1 | 189.67 | 23.64 | 223.65 | 15 | 184.97 | 4.22 |
| E | kbe_2 | | *P lobata* | pigmented | 2 | 183.72 | 17.82 | 219.81 | 16 | | |
| E | kbe_2 | | *P lobata* | pigmented | 3 | 181.52 | 19.21 | 217.02 | 11 | | |
| E | kbe_3 | 27 | *P lobata* | bleached | 1 | 92.15 | 6.56 | 98.22 | 18 | 118.01 | 22.40 |
| E | kbe_3 | | *P lobata* | bleached | 2 | 131.41 | 24.83 | 174.06 | 8 | | |
| E | kbe_3 | | *P lobata* | bleached | 3 | 130.48 | 11.12 | 140.16 | 7 | | |
| E | kbe_3 | 27 | *P lobata* | pigmented | 1 | 233.87 | 28.32 | 264.18 | 7 | 218.99 | 17.16 |
| E | kbe_3 | | *P lobata* | pigmented | 2 | 222.89 | 12.07 | 240.09 | 10 | | |
| E | kbe_3 | | *P lobata* | pigmented | 3 | 200.22 | 24.61 | 234.12 | 9 | | |
| F | kbf_2 | 28 | *P damicornis* | pigmented | 1 | 4.02 | 0.75 | 4.67 | 5 | 18.44 | 14.9 |
| F | kbf_2 | | *P damicornis* | pigmented | 2 | 18.82 | 1.41 | 20.98 | 7 | | |
| F | kbf_2 | | *P damicornis* | pigmented | 3 | 38.87 | 1.15 | 48.59 | 10 | | |
| F | kbf_2 | | *P damicornis* | pigmented | 4 | 12.07 | 1.07 | 16.21 | 20 | | |
| F | kbf_4 | 30 | *P lobata* | pigmented | 1 | 75.53 | 8.61 | 93.15 | 28 | 61.03 | 11.72 |
| F | kbf_4 | | *P lobata* | pigmented | 2 | 48.98 | 7.75 | 62.04 | 32 | | |
| F | kbf_4 | | *P lobata* | pigmented | 3 | 54.65 | 7.68 | 67.17 | 16 | | |
| F | kbf_4 | | *P lobata* | pigmented | 4 | 64.96 | 6.21 | 80.8 | 41 | | |
| F | kbf_5 | 31 | *P lobata* | bleached | 1 | 53.05 | 3.94 | 63.45 | 38 | 60.71 | 8.27 |
| F | kbf_5 | | *P lobata* | bleached | 2 | 62.07 | 3.42 | 68.34 | 12 | | |
| F | kbf_5 | | *P lobata* | bleached | 3 | 71.76 | 9.12 | 86.63 | 13 | | |
| F | kbf_5 | | *P lobata* | bleached | 4 | 55.96 | 3.06 | 61.7 | 10 | | |
| F | kbf_5 | 31 | *P lobata* | pigmented | 1 | 56.04 | 4.61 | 66.48 | 48 | 52.86 | 2.83 |
| F | kbf_5 | | *P lobata* | pigmented | 2 | 51.9 | 5.12 | 62.28 | 15 | | |
| F | kbf_5 | | *P lobata* | pigmented | 3 | 50.64 | 5.54 | 63.45 | 39 | | |

**Supplementary Table 2. Diel superoxide data from *Porites compressa*.**

| Health State | Time of Day | PAR | SW Normalized Superoxide (nM) | | |
|---|---|---|---|---|---|
| | | μmol $m^{-2}$ $s^{-1}$ | Average | SD | n |
| Bleached | 1230 | 1109 | 40.2 | 5.7 | 73 |
| Bleached | 1800 | 8.031 | 29.3 | 4.8 | 63 |
| Bleached | 2100 | 0 | 23.6 | 6.5 | 124 |
| Bleached | 500 | 0 | 27.4 | 4.0 | 69 |
| Bleached | 1100 | 1032 | 26.4 | 5.8 | 79 |
| Bleached | 1130 | 329 | 30.0 | 6.1 | 21 |
| Pigmented | 1230 | 1109 | 55.0 | 5.7 | 71 |
| Pigmented | 1800 | 8.031 | 37.7 | 6.2 | 48 |
| Pigmented | 2100 | 0 | 26.4 | 5.7 | 98 |
| Pigmented | 500 | 0 | 45.1 | 6.3 | 101 |
| Pigmented | 1100 | 1032 | 50.1 | 23.2 | 118 |
| Pigmented | 1130 | 329 | 39.8 | 15.7 | 74 |

**Supplementary Table 3.** ***Symbiodinium* counts**. Coral number corresponds to the same samples listed in Table S1. Replicate *Symbiodinium* counts from different coral fragments typically agreed to within ± 5% (SD).

| Coral No. | Category | Species | *Symbiodinium* density (cells cm$^{-2}$) |
|---|---|---|---|
| 1 | bleached | *Porites compressa* | 8.41E+04 |
| 2 | pigmented | *Porites compressa* | 3.08E+06 |
| 3 | bleached | *Montipora capitata* | 2.46E+05 |
| 4 | pigmented | *Montipora capitata* | 4.80E+05 |
| 5 | bleached | *Porites compressa* | 7.20E+05 |
| 6 | pigmented | *Porites compressa* | 3.08E+06 |
| 7 | bleached | *Porites compressa* | 2.76E+04 |
| 8 | pigmented | *Porites compressa* | 9.18E+05 |
| 9 | bleached | *Porites compressa* | 8.61E+05 |
| 9 | bleached | *Porites compressa* | 4.27E+05 |
| 10 | pigmented | *Montipora capitata* | 6.67E+05 |
| 11 | pigmented | *Montipora capitata* | 3.69E+05 |
| 12 | bleached | *Montipora capitata* | 2.24E+04 |
| 13 | pigmented | *Pocillopora damicornis* | 6.57E+04 |
| 13 | bleached | *Pocillopora damicornis* | 2.16E+03 |
| 17 | pigmented | *Pocillopora damicornis* | 1.23E+05 |
| 17 | bleached | *Pocillopora damicornis* | 9.89E+03 |
| 18 | bleached | *Pocillopora damicornis* | 4.06E+03 |
| 19 | pigmented | *Pocillopora damicornis* | 5.35E+04 |
| 19 | bleached | *Pocillopora damicornis* | 1.24E+03 |
| 20 | pigmented | *Montipora capitata* | 1.32E+06 |
| 21 | pigmented | *Porites compressa* | 1.40E+06 |
| 22 | bleached | *Montipora capitata* | 5.71E+04 |
| 25 | bleached | *Porites compressa* | 3.25E+05 |
| 26 | pigmented | *Porites lobata* | 1.30E+06 |
| 26 | bleached | *Porites lobata* | 4.39E+04 |
| 27 | pigmented | *Porites lobata* | 2.91E+06 |
| 27 | pigmented | *Porites lobata* | 2.62E+06 |
| 27 | bleached | *Porites lobata* | 8.69E+04 |
| 28 | pigmented | *Pocillopora damicornis* | 3.84E+04 |
| 29 | pigmented | *Pocillopora damicornis* | 1.42E+05 |
| 30 | pigmented | *Porites lobata* | 1.96E+06 |
| 31 | pigmented | *Porites lobata* | 3.00E+06 |
| 31 | bleached | *Porites lobata* | 2.56E+05 |

**Supplementary Table 4: Microbiome results.** Mean relative abundance and taxonomic affiliation (to family or genus level) of the bacterial community members associated with bleached and pigmented colonies of each coral species (note: no archaeal sequences were detected at this level).

| Abund. (%) | Taxonomic affiliation | Abund. (%) | Taxonomic affiliation |
|---|---|---|---|
| ***Pocillopora damicornis* bleached (n=3)** | | ***Pocillopora damicornis* pigmented (n=3)** | |
| 13.58 | Planctomycetes, Planctomycetacia, Planctomycetales, Planctomycetaceae, Pir4 lineage | 15.33 | Planctomycetes, Planctomycetacia, Planctomycetales, Planctomycetaceae |
| 5.00 | Planctomycetes, Planctomycetacia, Planctomycetales, Planctomycetaceae, Planctomyces | 2.62 | Planctomycetes, Planctomycetacia, Planctomycetales, Planctomycetaceae, Blastopirellula |
| 5.24 | Planctomycetes, Planctomycetacia, Planctomycetales, Planctomycetaceae, Rhodopirellula | 3.11 | Planctomycetes, Planctomycetacia, Planctomycetales, Planctomycetaceae, Pir4 lineage |
| 0.93 | Planctomycetes, Planctomycetacia, Planctomycetales, Planctomycetaceae, Rubripirellula | 11.59 | Planctomycetes, Planctomycetacia, Planctomycetales, Planctomycetaceae, Pirellula |
| 7.56 | Planctomycetes, Planctomycetacia, Planctomycetales, Planctomycetaceae, Blastopirellula | 7.61 | Planctomycetes, Planctomycetacia, Planctomycetales, Planctomycetaceae, Rhodopirellula |
| 8.23 | Planctomycetes, Planctomycetacia, Planctomycetales, Planctomycetaceae | 1.28 | Planctomycetes, Planctomycetacia, Planctomycetales, Planctomyce, Planctomyces |
| 2.21 | Planctomycetes, Planctomycetacia, Planctomycetales, Planctomyce, Planctomyces | 10.00 | Cyanobacteria, Cyanobacteria, SubsectionIV, FamilyII, Calothrix |
| 11.93 | Verrucomicrobia, Verrucomicrobiae, Verrucomicrobiales, Rubritaleaceae, Rubritalea | 0.95 | Cyanobacteria, Cyanobacteria, SubsectionI, FamilyI, Synechococcus |
| 10.43 | Cyanobacteria, Cyanobacteria, SubsectionIV, FamilyII, Calothrix | 1.41 | Cyanobacteria, Cyanobacteria, SubsectionII, FamilyII, Pleurocapsa |
| 0.55 | Cyanobacteria, Cyanobacteria, SubsectionIII, FamilyI | 3.24 | Cyanobacteria, Cyanobacteria, SubsectionIII, FamilyI |
| 6.66 | Proteobacteria, Gammaproteobacteria, Cellvibrionales, Spongiibacteraceae, BD1-7 clade | 9.11 | Proteobacteria, Alphaproteobacteria, Rhizobiales, Hyphomicrobiaceae, Filomicrobium |
| 0.84 | Proteobacteria, Gammaproteobacteria, Oceanospirillales, Halomonadaceae, Cobetia | 6.60 | Proteobacteria, Gammaproteobacteria, Oceanospirillales, Hahellaceae, Endozoicomonas |
| 3.59 | Firmicutes, Clostridia, Clostridiales, Clostridiaceae 1, Clostridium sensu stricto 1 | 4.21 | Proteobacteria, Gammaproteobacteria, Cellvibrionales, Microbulbiferaceae, Microbulbifer |
| 2.35 | Firmicutes, Clostridia, Clostridiales, Peptostreptococcaceae | 2.33 | Proteobacteria, Gammaproteobacteria, Pseudomonadales, Moraxellaceae, Psychrobacter |
| 2.05 | Actinobacteria, Acidimicrobiia, Acidimicrobiales, Acidimicrobiales Incertae Sedis, Candidatus Microthrix | 0.52 | Proteobacteria, Deltaproteobacteria, Bdellovibrionales, Bdellovibrionaceae, Bdellovibrio |
| 2.09 | Chloroflexi, Chloroflexia, Chloroflexales, Roseiflexaceae, Roseiflexus | 1.36 | Verrucomicrobia, Verrucomicrobiae, Verrucomicrobiales, Rubritaleaceae, Rubritalea |
| 2.06 | Deinococcus-Thermus, Deinococci, Deinococcales, Trueperaceae, Truepera | 0.79 | Firmicutes, Clostridia, Clostridiales, Clostridiaceae 1, Clostridium sensu stricto 1 |
| 0.65 | Proteobacteria, Alphaproteobacteria, Rhizobiales, Rhodobiaceae, Methyloceanibacter | 0.87 | Firmicutes, Clostridia, Clostridiales, Lachnospiraceae, Epulopiscium |
| 0.80 | Proteobacteria, Deltaproteobacteria, Desulfovibrionales, Desulfovibrionaceae, Desulfovibrio | 1.38 | Firmicutes, Clostridia, Clostridiales, Ruminococcaceae |
| | | 1.67 | Firmicutes, Clostridia, Clostridiales, Ruminococcaceae, Ruminiclostridium 1 |
| ***Porites compressa* bleached (n=3)** | | ***Porites compressa* pigmented (n=3)** | |
| 59.04 | Proteobacteria, Gammaproteobacteria, Oceanospirillales, Hahellaceae, Endozoicomonas | 35.67 | Proteobacteria, Gammaproteobacteria, Oceanospirillales, Hahellaceae, Endozoicomonas |
| 15.46 | Proteobacteria, Alphaproteobacteria, Rhodobacterales, Rhodobacteraceae, Pseudovibrio | 23.88 | Alphaproteobacteria, Sphingomonadales, Erythrobacteraceae |
| 7.69 | Firmicutes, Clostridia, Clostridiales, Clostridiaceae 4, Clostridium sensu stricto | 1.24 | Alphaproteobacteria, Sphingomonadales, Erythrobacteraceae, Erythrobacter |
| 3.48 | Bacteroidetes, Sphingobacteriia, Sphingobacteriales, Saprospiraceae, Portibacter | 14.27 | Bacteroidetes, Cytophagia, Cytophagales, Flammeovirgaceae, Candidatus Amoebophilus |
| 2.35 | Proteobacteria, Alphaproteobacteria, Rhodospirillales, Rhodospirillaceae, | 7.59 | Actinobacteria, Actinobacteria, Corynebacteriales, Corynebacteriaceae, |

## **Supplementary Table 4** (continued)

| | | | |
|---|---|---|---|
| | Pelagibius | | Corynebacterium 1 |
| 0.62 | Proteobacteria, Deltaproteobacteria, Desulfobacterales, Nitrospinaceae, Candidatus Entotheonella | 3.80 | Proteobacteria, Deltaproteobacteria, Bdellovibrionales, Bdellovibrionaceae, Bdellovibrio |
| 1.02 | Proteobacteria, Gammaproteobacteria, Cellvibrionales, Halieaceae, OM60(NOR5) clade | 3.65 | Planctomycetes, Planctomycetacia, Planctomycetales, Planctomycetaceae |
| 1.50 | Proteobacteria, Alphaproteobacteria, Rhizobiales, Hyphomicrobiaceae, Filomicrobium | 0.31 | Planctomycetes, Planctomycetacia, Planctomycetales, Planctomycetaceae, Pir4 lineage |
| 1.21 | Proteobacteria, Alphaproteobacteria, Rhizobiales, Rhodobiaceae | 0.35 | Planctomycetes, Planctomycetacia, Planctomycetales, Planctomycetaceae, Rhodopirellula |
| 1.23 | Verrucomicrobia, Verrucomicrobiaea, Verrucomicrobiales, Rubritaleaceae, Rubritalea | 3.14 | Proteobacteria, Alphaproteobacteria, Parvularculales, Parvularculaceae, Parvularcula |
| 1.06 | Planctomycetes, Planctomycetacia, Planctomycetales, Planctomyce, Planctomyces | 1.16 | Firmicutes, Clostridia, Clostridiales, Lachnospiraceae, Epulopiscium |
| 0.57 | Planctomycetes, Planctomycetacia, Planctomycetales, Planctomycetaceae, Planctomyces | 0.42 | Proteobacteria, Gammaproteobacteria, Pseudomonadales, Moraxellaceae, Acinetobacter |
| | | 0.73 | Verrucomicrobia, Verrumicrobiae, Verrucomicrobiales, Rubritaleaceae, Rubritalea |
| ***Porites lobata*** **bleached (n=1)** | | ***Porites lobata*** **pigmented (n=2)** | |
| 70.58 | Alphaproteobacteria, Sphingomonadales, Erythrobacteraceae, Erythrobacter | 21.02 | Proteobacteria, Deltaproteobacteria, Desulfovibrionales, Desulfovibrionaceae, Desulfovibrio |
| 8.37 | Alphaproteobacteria, Sphingomonadales, Erythrobacteraceae | 16.08 | Alphaproteobacteria, Sphingomonadales, Erythrobacteraceae, Erythrobacter |
| 16.94 | Proteobacteria, Alphaproteobacteria, Caulobacterales, Hyphomonadaceae, Maricaulis | 18.61 | Gammaproteobacteria, Chromatiales, Chromatiaceae, Nitrosococcus |
| 4.12 | Proteobacteria, Gammaproteobacteria, Oceanospirillales, Hahellaceae, Endozoicomonas | 15.36 | Proteobacteria, Gammaproteobacteria, Vibrionales, Vibrionaceae, Photobacterium |
| | | 7.99 | Planctomycetes, Phycisphaerae, Phycisphaerales, Phycisphaeraceae |
| | | 7.47 | Actinobacteria, Actinobacteria, Corynebacteriales, Nocardiaceae, Rhodococcus |
| | | 5.36 | Planctomycetes, Planctomycetacia, Planctomycetales, Planctomyce, Planctomyces |
| | | 2.65 | Planctomycetes, Planctomycetacia, Planctomycetales, Planctomycetaceae, Pir4 lineage |
| | | 2.22 | Proteobacteria, Alphaproteobacteria, Sphingomonadales, Erythrobacteraceae, Erythrobacter |
| | | 1.94 | Proteobacteria, Gammaproteobacteria, Oceanospirillales, Hahellaceae, Endozoicomonas |
| | | 1.27 | Spirochaetae, Spirochaetes, Spirochaetales, Spirochaetaceae |
| | | 0.03 | Proteobacteria, Gammaproteobacteria, Enterobacteriales, Enterobacteriaceae, Escherichia-Shigella |
| ***Montipora capitata*** **bleached (n=1)** | | ***Montipora capitata*** **pigmented (n=1)** | |
| 36.64 | Verrucomicrobia, Verrucomicrobiae, Verrucomicrobiales, Rubritaleaceae, Rubritalea | 100.00 | Proteobacteria, Alphaproteobacteria, Rhodobacterales, Rhodobacteraceae |
| 2.40 | Verrucomicrobia, Opitutae, Puniceicoccales, Puniceicoccaceae, Puniceicoccus | | |
| 25.85 | Planctomycetes, Planctomycetacia, Planctomycetales, Planctomycetaceae, Pir4 lineage | | |
| 12.45 | Planctomycetes, Planctomycetacia, Planctomycetales, Planctomycetaceae, Rhodopirellula | | |
| 7.76 | Planctomycetes, Planctomycetacia, Planctomycetales, Planctomyce, Schlesneria | | |
| 2.01 | Planctomycetes, Planctomycetacia, Planctomycetales, Planctomyce, Planctomyces | | |

**Supplementary Table 4** (continued)

| | | |
|---|---|---|
| 7.76 | Cyanobacteria, Cyanobacteria, SubsectionII, FamilyII, Pleurocapsa | |
| 2.43 | Proteobacteria, Gammaproteobacteria, Order Incertae Sedis, Family Incertae Sedis, Marinicella | |
| 0.56 | Proteobacteria, Alphaproteobacteria, Sphingomonadales, Erythrobacteraceae, Erythrobacter | |
| 0.53 | Proteobacteria, Alphaproteobacteria, Rhizobiales, Phyllobacteriaceae, Cohaesibacter | |

**Supplementary Table 5. Extracellular superoxide production by coral bacteria and coral larvae**

| Organism | Superoxide production ($amol\ individual^{-1}\ hr^{-1}$) Avg | SD | n |
|---|---|---|---|
| *Endozoicomonas montiporae* LMG-24815 | 0.13 | 0.06 | 3 |
| *Ruegeria* sp. WHOICORAL16 | 1.1 | 0.1 | 3 |
| *Vibrio* sp. WHOICORAL50 | 2.2 | 1.2 | 3 |
| *Diploria labyrinthiformis* | $6.0 \times 10^5$ | $9.6 \times 10^4$ | 2 |
| *Orbicella faveolata* | $8.2 \times 10^5$ | $5.9 \times 10^5$ | 2 |
| *Colpophyllia natans* | $2.3 \times 10^6$ | $4.5 \times 10^5$ | 2 |
